# Supplementary material for: Content-rich biological network constructed by mining PubMed abstracts
Source: BMC Bioinformatics. 2004 Oct 8;5:147. doi: 10.1186/1471-2105-5-147 (PMC528731; doi:10.1186/1471-2105-5-147)
Supplement: Additional File 2 — The original results of the above study (non-essential files are deleted to keep the file size under the limit set by BMC bioinformatics). [file 1471-2105-5-147-S2.bz2 › chilibotAdditionalFile2/dip10/415ID10504300E10081/html/BMPR1A_BMP6.html]

 


 **BMPR1A** and **BMP6** 
  
Found 5 abstracts in PubMed, retrieved 5.  
 

 What does Google say? 
 PDF only 
| .edu only 

---

**Interactive relationship** (e.g. stimulation, inhibition, etc)

**Neutral relationship**- In addition, BMP 6  [ **BMP6** ]  weakly bound to BMPR IA ALK 3  [ **BMPR1A** ] , to which BMP 2 also bound.  Ref: 10504300 J Cell Sci, 1999
